# The South African Rugby Injury and Illness Surveillance and Prevention Project (SARIISPP): SARU Girls’ Youth Week Injury Surveillance Report, 2019

**DOI:** 10.17159/2078-516X/2021/v33i1a12487

**Published:** 2021-01-15

## Executive Summary

As part of the South African Rugby Injury and Illness Surveillance and Prevention Project (SARIISPP), the annual SARU Youth Week tournaments’ injury data are recorded and investigated by SA Rugby. The BokSmart National Rugby Safety Programme has been collecting and analysing these data annually since 2011 for the SARU Boy’s Youth Week tournaments. In 2015, the SARU Girls’ Youth Week data collection began; this report being the first to analyse the Girls’ Youth Week data.

The analysis shows injury patterns over time between tournaments, and collectively, for the girls’ u16 and u18 SARU Youth Weeks. Additionally, the analysis compares the profiles of injured players at each individual tournament. When investigating these patterns, areas of concern are identified, changes in the game, tournament structure or medical support services are considered or contested against the evidence, and injury specific interventions can be created and implemented, where the evidence indicates such a need.

Each medical facility at the SARU Youth Week tournaments has a designated researcher onsite, who together with the tournament medical doctors, records the tournament injury data daily. Three injury cases were removed from the analysis. These data were recorded but did not appear to be accurate on follow-up and were therefore removed. Unfortunately, inaccuracy can occur during data collection and measures have been implemented to ensure that this is limited.

This 2019 SARU Girls’ Youth Week report focuses on the Girls’ tournaments, comprising of the Girls u16 Week (Gu16W) and Girls u18 Week (Gu18W) held in 2019. The tournaments consisted of 32 teams and 48 matches. Comparisons are made between SARU Girls’ Youth Week tournaments and over time between 2015 and 2019. It must be noted that no Gu16W tournament was held in 2017.

In 2019, the Gu16W recorded a higher Time-Loss injury incidence at 22 (11–33) [mean (95% confidence intervals)] injuries per 1000 player hours. Gu18W recorded slightly lower Time-Loss injury incidence at 19 (10 – 28) injuries per 1000 player hours. The collective tournament average was measured at 21 (13 to 28) injuries per 1000 player hours. When combining the injury incidence data collected over the five years, Gu18W had a lower Time-Loss injury incidence.

In 2019, the Tackler and Open Play, followed by the Ball Carrier, were the most frequent injury-causing events in that order. *Tackling front-on (regulation)*, *Tackling LOW side-on*, and *Tackling LOW front-on*, were the most frequent injury causing mechanisms involved in the Tackler phases of play. While *Collision in Open Play* was the most frequent injury causing mechanism in Open Play.

The most common injury type was Central Nervous System injuries, where Gu18W recorded a higher incidence. Head and Neck were the most common injury locations in 2019, accounting for 69% of the injuries, with most of these injuries occurring in the Gu18W. Scrumhalves and flyhalves were the player positions with the highest normalised injury incidence per player per position across all tournaments.

As expected, the injury incidence of *‘New’* injuries was higher than subsequent ‘*Recurrent’* injuries. The majority of ‘*New*’ injuries were injuries to the joint, while most *‘Recurrent’* injuries were ligament and joint injuries.

Fourteen concussions occurred across the two tournaments in 2019, which has dropped since the spike recorded in 2018. The Gu18W had the higher concussion incidence of the two tournaments. Furthermore, the act of *Tackling* contributed to 50% of the events causing concussions.

The tackle contest is clearly an event that requires additional injury prevention focus for coaches on preparing their younger female players better for rugby, and requires more time spent on teaching them safer techniques and body positions in the tackle contest.

## Definitions

All definitions are originally based on the 2007 consensus statement for injury reporting in rugby union (1) and have since been realigned with the latest International Olympic Committee (IOC) consensus statement for methods of recording and reporting epidemiological data on injury and illness in sport (2–3).

### MEDICAL ATTENTION INJURY

Injury, according to the International Olympic Committee Consensus Statement of 2020, can be defined as *“tissue damage or other derangement of normal physical function due to participation in sports, resulting from rapid or repetitive transfer of kinetic energy”* (2). All injuries managed by the Tournament Medical Doctors were classified as Medical Attention injuries. These are defined by the 2007 statement as an “*injury that results in a player receiving medical attention”* (1), and by the more recent IOC statement as *“a health problem that results in an athlete receiving medical attention”* (2–3).

### TIME-LOSS INJURY

Medical Attention injuries were further categorised as Time-Loss injuries, where appropriate, and defined by the 2007 statement as, “*an injury that results in a player being unable to take a full part in future rugby training or match play*” (1). The IOC definition is, *“a health problem that results in a player being unable to complete the current or future training session or competition”* (2). In this report it is specific to injuries (3).

### INJURY RATE

For this 2019 SARU Girls’ Youth Week report, an injury rate is the number of injuries expressed per 1000 player exposure hours. This normalised version of the number of injuries has been used in previous reports and enables comparison between current tournaments, previous tournaments and to other published scientific literature. Moreover, the injury rate is expressed as a mean with 95% confidence intervals. A 95% confidence interval around a mean value indicates that we can be 95% certain that the value is bounded by the two intervals. In this report, we present the 95% confidence intervals assuming normal distribution of the data and use the approach of examining the overlap of the confidence intervals to determine whether the injury incidences are significantly different. If the range of confidence interval values of two comparisons do not overlap, there is a strong (95%) chance that their injury rates are different from each other. This method is conservative and is less likely to produce false positive results (3–4).

### NEW, SUBSEQUENT AND RECURRENT INJURIES

In the 2019 SARU Girls’ Youth Week report, a ‘*New Injury’* was defined as when a player sustained her first injury in the tournament. Any injury that the *same* player sustained after this initial injury was defined as a *‘Subsequent Injury’.*

According to the more recent IOC statement, any subsequent injury to the same site and of the same type is referred to as a ‘*Recurrence’* if the index injury was fully recovered before reinjury, and as an *‘Exacerbation’* if the index injury was not yet fully recovered (2).

To provide more detail on the subsequent injuries for practitioners, we have further categorized the subsequent injuries in this report into one of four groups based on the OSICS classification diagnosis (5):

- Different site - Different type
- Different site - Same type
- Same site - Different type
- Same site - Same type

According to the 2007 Consensus Statement for rugby, and due to the timing and nature of the tournament, any subsequent injury classified as ‘Same site -Same type’ was classified as a *‘Recurrent injury’* (1, 3).[Fig f23-2078-516x-33-v33i1a12487]

**Figure f23-2078-516x-33-v33i1a12487:**
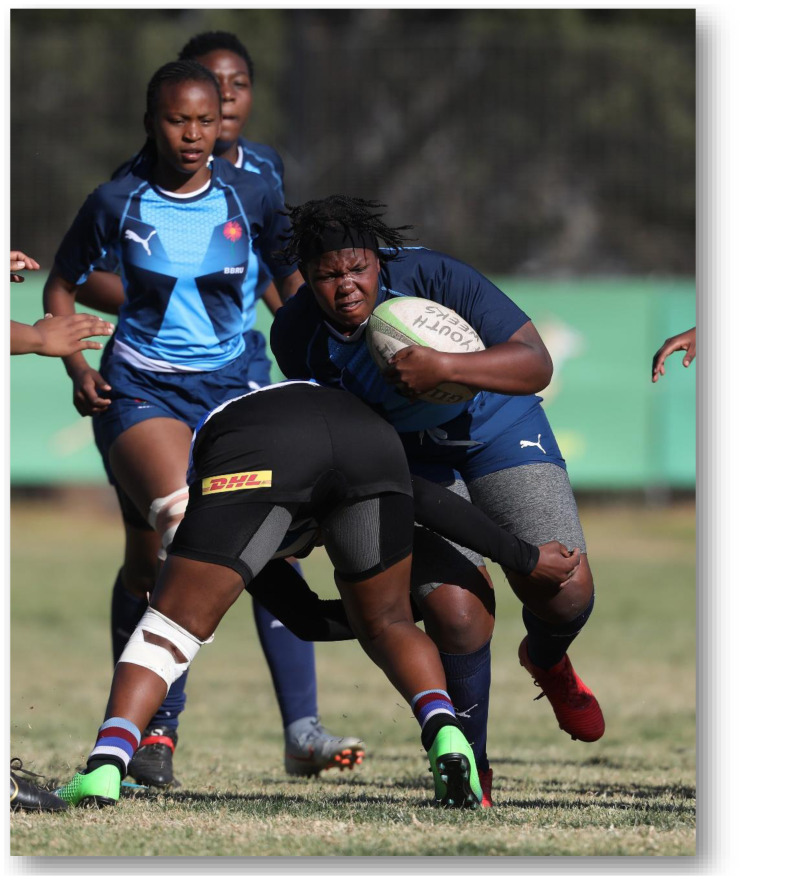


## Key Findings

### Injury Incidence

**Figure 1:**
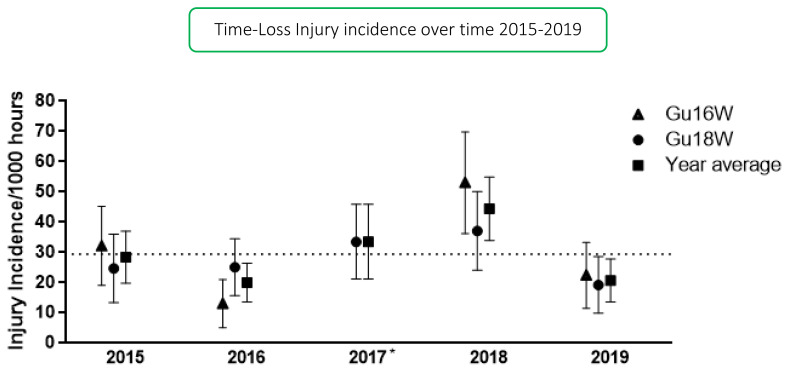
Injury incidence/1000 player hours and 95% confidence intervals of Time-Loss injuries for the SARU Girls’ Youth Week Tournaments from 2015–2019. The dotted line reflects the average incidence for all tournaments over all of the included years. *No Gu16W tournament was held in 2017.

In 2019, thirty-two teams participated in the SARU Girls’ Youth Week tournaments (Gu16W = 16 teams, Gu18W = 16 teams). A total of 171 Medical Attention injuries were recorded during the tournaments; nineteen percent of these (n = 32) were Time-Loss injuries. The combined tournaments’ injury incidence and 95% confidence intervals for all Medical Attention injuries was 110 (93 to 126) injuries/1000 player hours, and for Time-Loss injuries was 21 (13 to 28) injuries/1000 player hours. There was no significant difference between the two SARU Girls’ Youth Week tournaments in 2019 (Table 1). The numbers of Medical Attention and Time-Loss injuries per match and per hour of match play across the tournaments are represented in Table 2. Figure 1 shows the pattern of Injury incidence/1000 player hours and 95% confidence intervals of Time-Loss injuries for each tournament across the years (2015 to 2019). It must be noted that the Gu16W tournament in 2017 did not take place (Figure 1).

Three injury cases were removed from the analysis. These data were initially captured, however, could not be verified and did not appear to be accurate on follow-up, and were therefore removed from further analysis. Partial details were missing from a number of cases throughout the data collection process. All these cases are highlighted throughout the report for transparency. Measures have been included in the standard operating procedures for data collection at future tournaments to improve the quality of the data.

**Figure 2:**
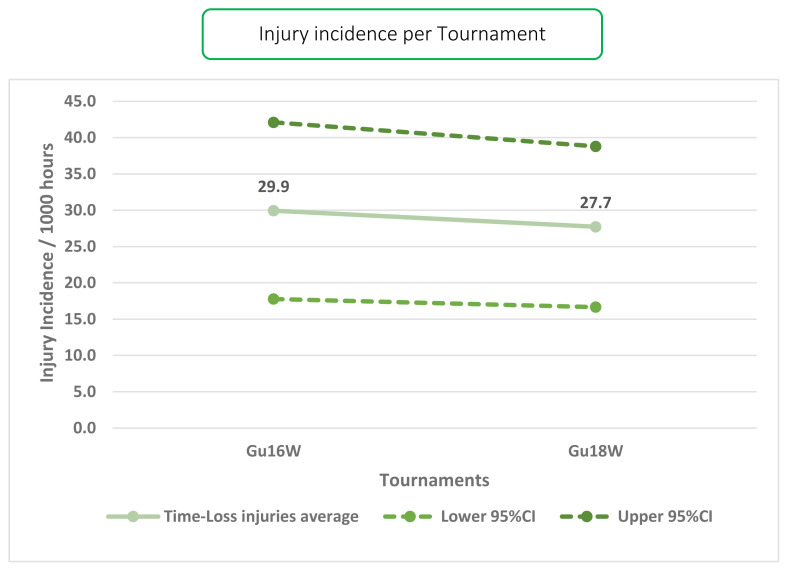
Injury incidence/1000 player hours and 95% confidence intervals (dotted lines) at the SARU Girls’ Youth Week tournaments from 2015–2019.

Only Time-Loss injuries were analysed further. Combined data from 2015 to 2019 shows a slight decrease in injury incidence from Gu16W to Gu18W (Figure 2). Overall, there is only a small and insignificant difference in injury incidence rates between these two tournaments.

### Injury Incidence Trends

#### Girls U16 Week (Gu16W)

**Figure 3:**
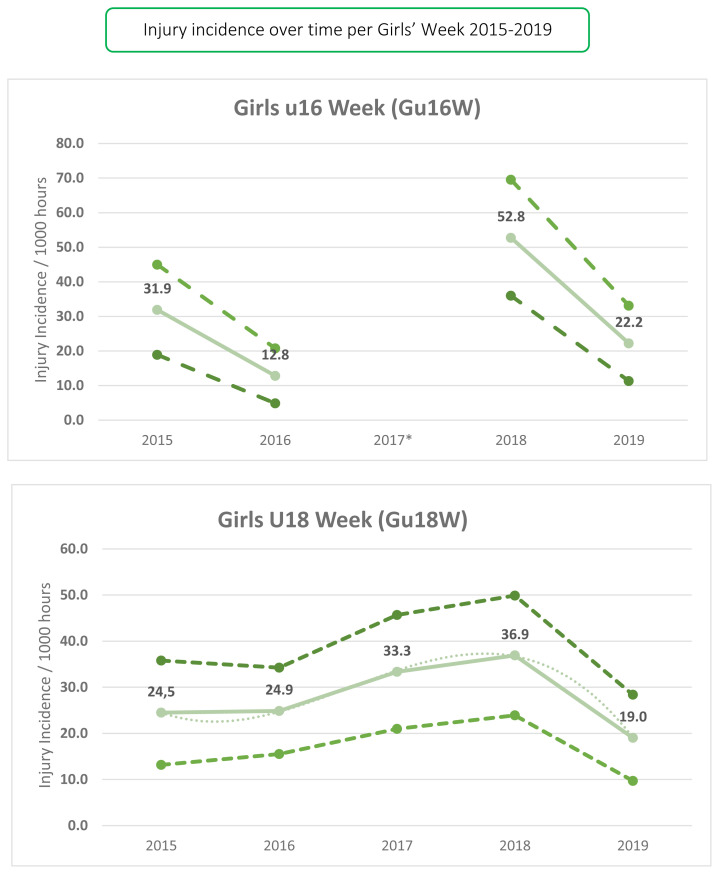
Time-Loss Injury incidence for each SARU Girls’ Youth Week tournament, per year, from 2015 – 2019, including the upper and lower 95% Confidence Intervals (95%CI). The dashed grey line in the Gu18W graph represents the polynomial trend. *No Gu16W tournament was held in 2017.

The Gu16W tournament was not held in 2017, so the trendline could not be calculated with much accuracy and has therefore been excluded. The highest injury incidence occurred in 2018, where there was a spike. In 2019, the Gu16W injury incidence decreased again to its second lowest rate recorded over the 4 years examined; 2017 was excluded due to no tournament being held (Figure 3).

#### Girls U18 Week (Gu18W)

There was a gradual increase in injury incidence at the Gu18W between 2015 and 2018 and then a slight decrease in 2019 (Figure 3). The injury incidence in 2019 was the lowest in the 5 years of the study. In Figure 3, the polynomial trendline accounts for 100% of the variance in injury incidence per 1000 player hours (R^2^ = 1.0). The trendline increases throughout the initial years, and then moves downward from 2018 to 2019.[Fig f24-2078-516x-33-v33i1a12487]

**Figure f24-2078-516x-33-v33i1a12487:**
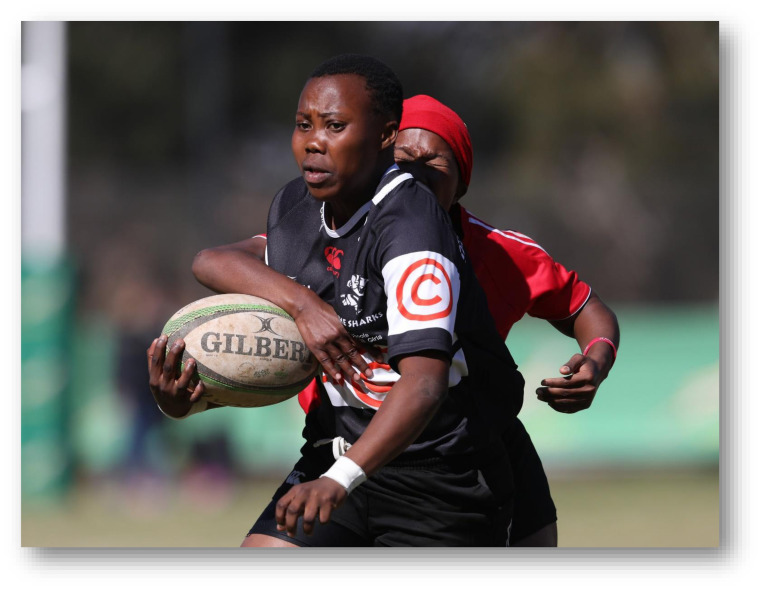


### Injury Event

In 2019, the Tackler role was the event associated with the most injuries throughout the tournaments (34%), followed by Open Play (31%) and the Ball Carrier (16%). Tacklers had 7 (3 to 11) injuries/1000 player hours, while injuries in Open Play had an injury incidence of 6 (2 to 10) injuries/1000 player hours and Ball Carriers 3 (0 to 6) injuries/1000 player hours.[Fig f25-2078-516x-33-v33i1a12487]

**Figure f25-2078-516x-33-v33i1a12487:**
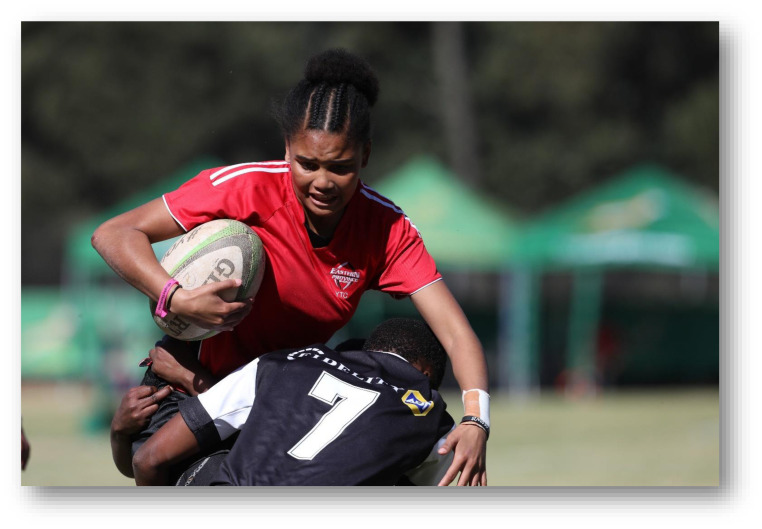


Injury incidence to the Tackler was higher in the Gu16W tournament, while Open Play injury incidence was higher in the Gu18W (Table 3).

**Figure 4:**
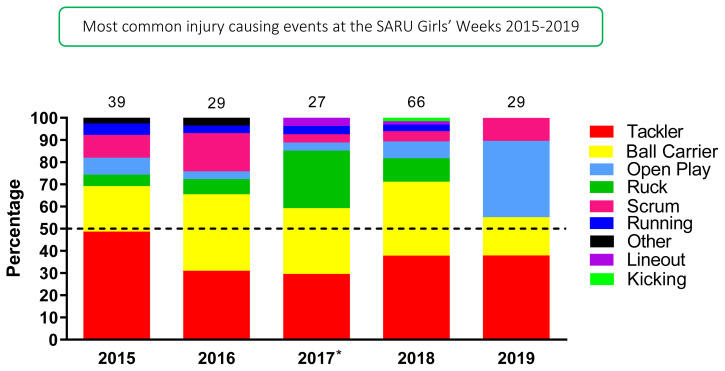
Most common injury causing events in the SARU Girls’ Youth Week tournaments from 2015–2019. (The number above each bar represents the total number of injuries for that year). Missing 2019 data = 3 cases. *No Gu16W tournament was held in 2017.

Figure 4 displays the grouped proportionate breakdown of injuries resulting from the different injury causing events between 2015 and 2019. The proportions of Tackler and Ball Carrier injuries fluctuated throughout the years but have remained the most prominent events causing injury. The Tackle event (both Tackler and Ball carrier combined) ranged between 55%–71% (averaging 64%) of all injuries per year in the Girls’ Weeks (both Gu16W and Gu18W) during this period. The tackle contest is clearly an event that requires additional injury prevention focus for coaches to prepare their female players better for rugby. More time is required in training to teach the players safer techniques and body positions in the tackle contest. There was an increase in injuries from Open Play in 2019.

**Figure 5:**
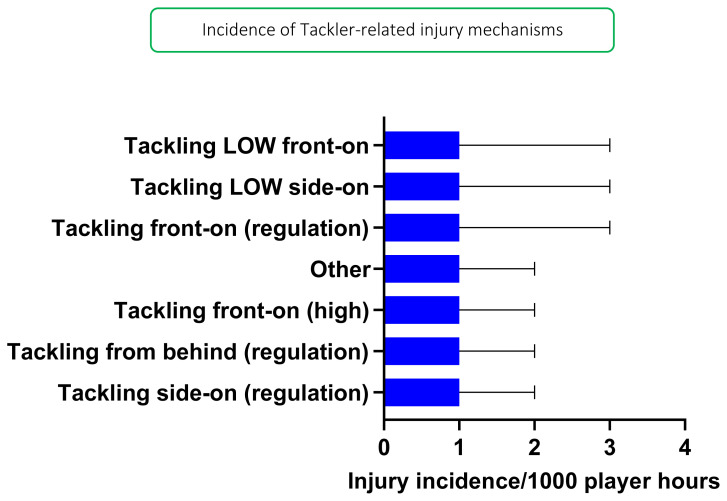
Injury incidence and 95% confidence intervals/1000 player hours of Tackler-related injury mechanisms at the 2019 SARU Girls’ Youth Week Tournaments. Missing 2019 data = 1 case.

**Figure 6:**
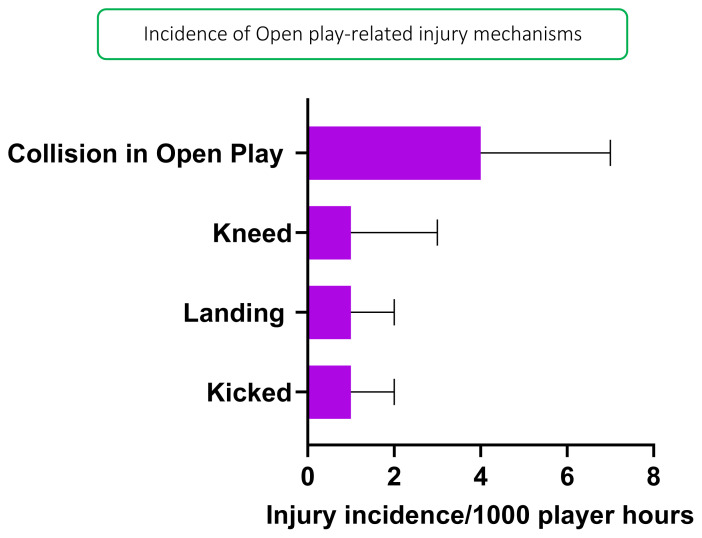
Injury incidence and 95% confidence intervals/1000 player hours for Open Play-related injury mechanisms at the 2019 SARU Girls’ Youth Week Tournaments.

In 2019, *Tackling front-on (regulation)*, *Tackling LOW side-on*, and *Tackling LOW front-on* were mechanisms that accounted equally for injuries to Tacklers (all at 18%) with 1 (0 to 3) injuries/1000 player hours (Figure 5). *Collision in Open Play* accounted for the highest proportion of Open Play injuries (60%) with 4 (0 to 7) injuries/1000 player hours (Figure 6).

**Figure 7:**
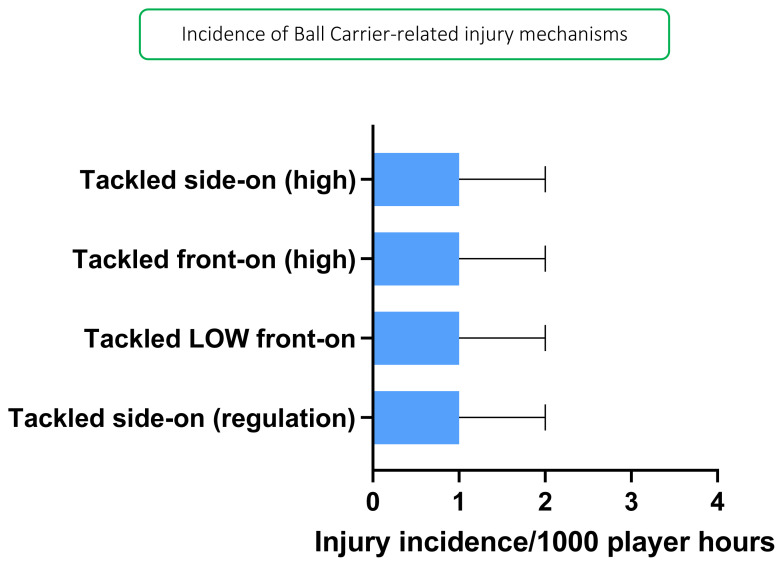
Injury incidence and 95% confidence intervals/1000 player hours of Ball Carrier-related injury mechanisms at the 2019 SARU Girls’ Youth Week Tournaments. Missing 2019 data = 1 case.

*Tackled LOW front-on*, *Tackled side-on (high)*, *Tackled front-on (high)* and *Tackled side-on (regulation)* accounted equally for injuries to the Ball Carrier in 2019 (all at 20%), with an injury incidence of 1 (0 to 2) injury/1000 player hours each (Figure 7).

### Injury Type

In 2019, CNS (Central Nervous System) injuries were the most common injury type (Table 4). There were significantly more CNS injuries than Muscle/Tendon injuries at the Gu16W tournament and in the combined data across both tournaments. Gu18W had the most CNS injuries, while Gu16W had more Joint/Ligament injuries than Gu18W.

**Figure 8:**
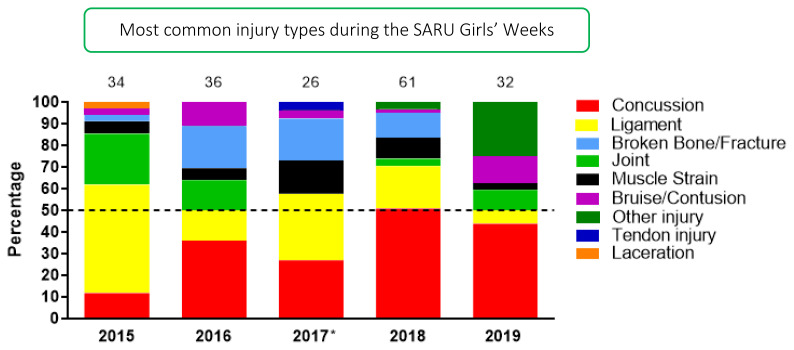
Most common injury types in the SARU Girls’ Youth Week tournaments from 2015–2019. (The number above each bar represents the total number of injuries for that year). *No Gu16W tournament was held in 2017.

Figure 8 shows the most common injury types in proportionate contribution per year from 2015 to 2019. Concussions and ligament injuries have decreased from 2018 to 2019 but were still the two most prominent injury types in the SARU Girls’ tournaments over the years. Concussions contributed to 51% and 44% of all injuries in 2018 and 2019, respectively. Bruise/Contusion and Other Injuries increased in 2019.

### Body Location

Injuries were grouped according to the four main body location groups (*Head and Neck; Trunk; Upper Body; Lower Body*) across all tournaments. In 2019, the most common injured body location was the *Head and Neck* (69%), with 64% of these injuries occurring at the Gu18W tournament. *Head and Neck* injuries accounted for an injury incidence of 14 (8 to 20) injuries/1000 player hours (Table 5). The Gu18W had the highest *Head and Neck* injury incidence recorded at 17 (8 to 25) injuries/1000 player hours, but this was not significantly different to the Gu16W.[Fig f26-2078-516x-33-v33i1a12487]

**Figure f26-2078-516x-33-v33i1a12487:**
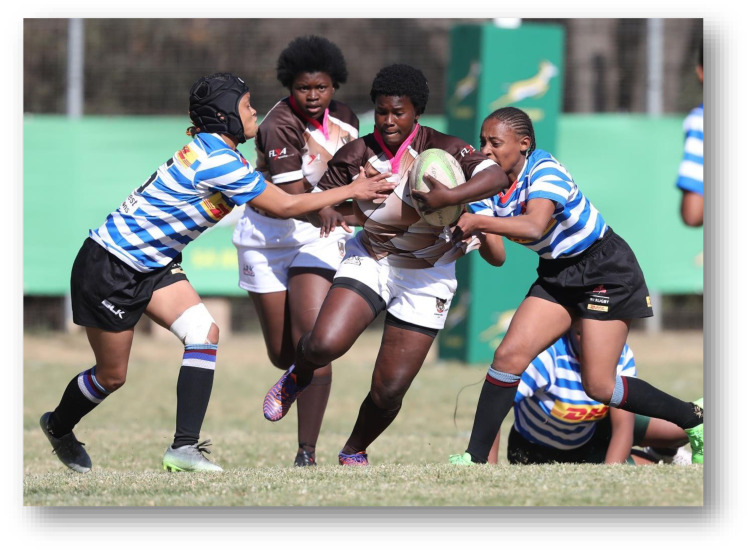


The recent IOC Consensus statement recommended categories of Tissue and Pathology injury data, are presented in Table 6 for the 2019 SARU Girls’ Youth Week tournaments (2). Due to six of the cases not being finalised properly and with operational follow-up notes not being received from the treating hospital emergency units, they have been classified as ‘Other injury’, with a broader description of their preliminary case notes included in the ‘Other injury’ category calculations. It can however be confirmed that none of them were serious injuries, and were all confirmed as minor, precautionary cases.

### New vs recurrent

The injury incidence of *‘New’* injuries in 2019 was 17 (11 to 24) injuries/1000 player hours; significantly higher than ‘*Recurrent’* injuries which had an injury incidence of 2 (0 to 4) injuries/1000 player hours.

**Figure 9:**
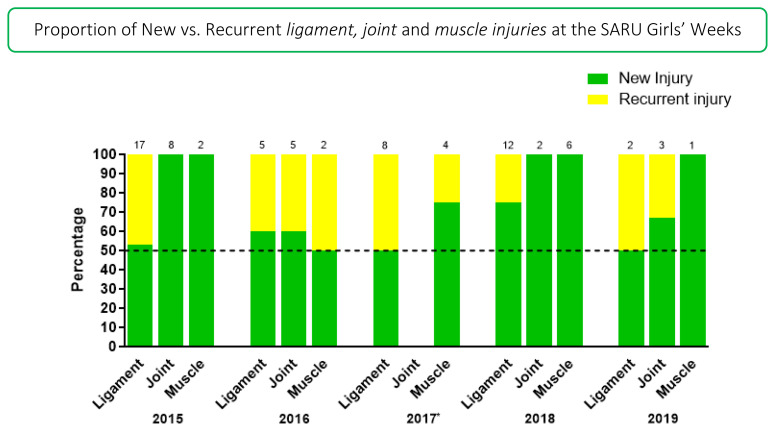
Proportion of New and Recurrent ligament, joint and muscle injuries in the SARU Girls’ Youth Week tournaments from 2015 – 2019. (The number above each bar represents the total number of injuries for that year). *No Gu16W tournament was held in 2017.

Figure 9 illustrates the proportion of *‘New’* and *‘Recurrent’* ligament, joint and muscle injuries across the years (2015–2019). The proportion of *‘New’* ligament injuries decreased from 2018 (75%) to 2019 (50%) and *‘New’* joint injuries from 2018 (100%) to 2019 (67%), while *‘New’* muscle injuries remained the same (100%).

‘*Recurrent’* ligament injuries increased from 2018 (25%) to 2019 (50%) and ‘*Recurrent’* joint injuries from 0% in 2018 to 33% in 2019. ‘*Recurrent’* muscle injuries remained the same (0%). The injury numbers in 2019 were however very low and therefore these changes need to be interpreted with caution.

### Game Quarter

**Figure 10:**
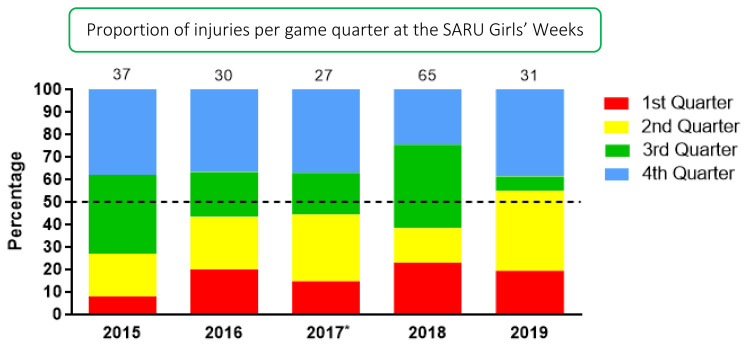
Proportion of injuries occurring in each game quarter in the SARU Girls’ Youth Week tournaments from 2015 – 2019. (The number above each bar represents the total number of injuries for that year). Missing data in 2019 = 1 case. *No Gu16W tournament was held in 2017.

In 2019, most injuries occurred in the 4^th^ quarter (39%) followed by the 2^nd^ quarter (35%) with an injury incidence of 8 (3 to 12) injuries/1000 player hours and 7 (3 to 11) injuries/1000 player hours respectively. There was a large increase from 2015 to 2019 in injuries in the 2^nd^ quarter, however this was not significant. There was a sizable decrease in injuries in the 3^rd^ quarter in 2019 compared to previous years (Figure 10).

### Player positions

**Figure 11:**
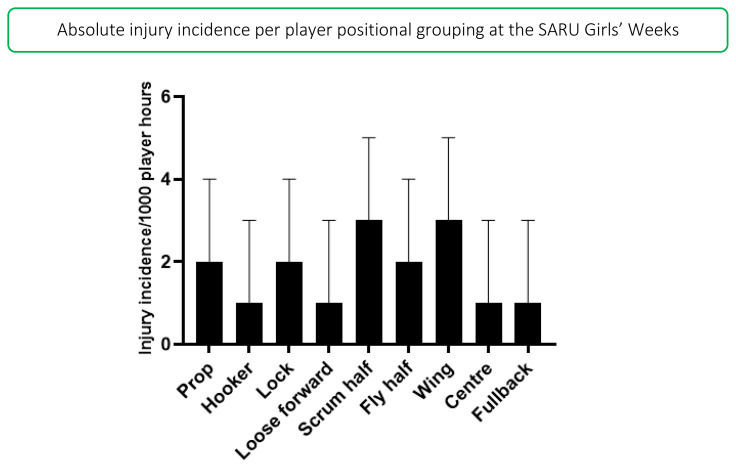
Absolute Injury incidence and 95% confidence intervals/1000 player hours per positional grouping in the SARU Girls’ Youth Week Tournaments 2019. Missing data in 2019 = 7 cases.

Absolute incidence refers to the incidence of injury in a player positional grouping, e.g., wings, without normalising for the number of players on the field playing in that positional grouping, e.g., there are two wings per team on the field. In 2019, the scrumhalf and wing positions had the highest absolute injury incidence rates across both tournaments. Scrumhalves and wings had an absolute injury incidence of 3 (0 to 5) injuries/1000 player hours (Figure 11).

**Figure 12:**
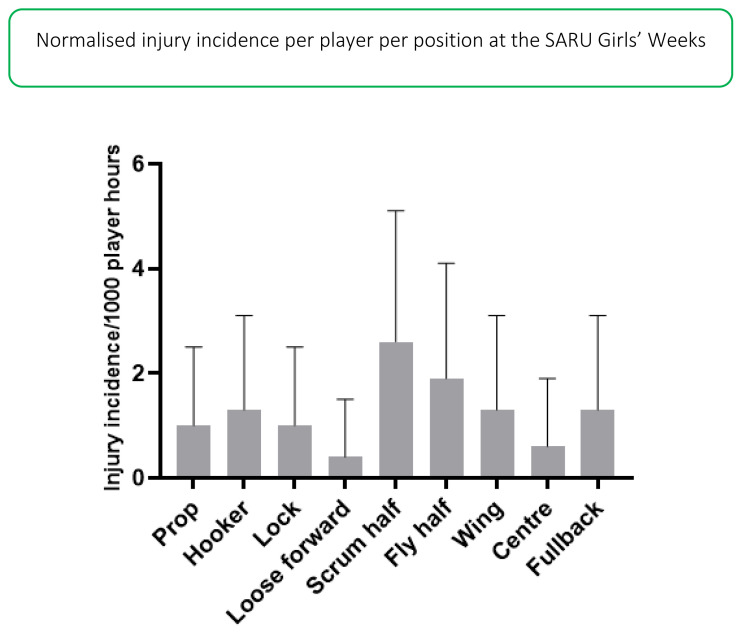
Normalised injury incidence and 95% confidence intervals/1000 player hours per player per position in the SARU Girls’ Youth Week Tournaments 2019. Missing data in 2019 = 7 cases.

**Figure 13:**
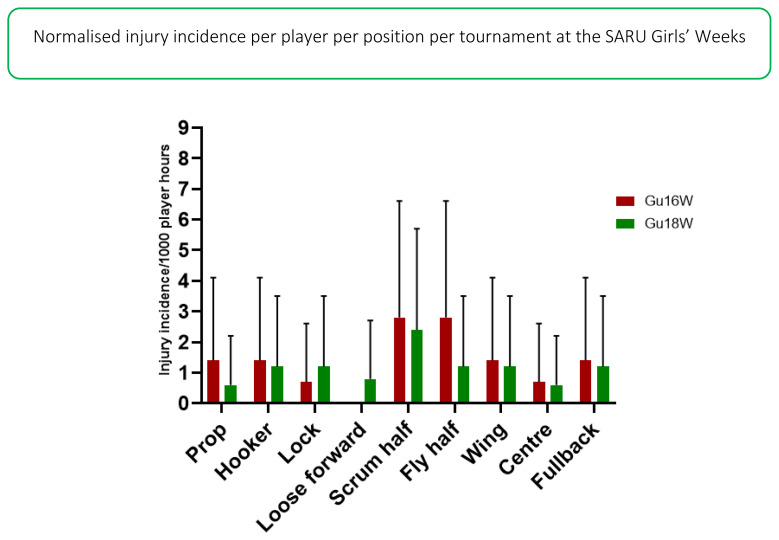
Normalised injury incidence and 95% confidence intervals/1000 player hours per player per position, across the two SARU Girls’ Youth Week Tournaments in 2019. Missing data in 2019 = 7 cases.

The number of injuries were also normalised to the number of players on the field in a positional grouping. For example: Props = total number of injuries divided by 2, Locks = total number of injuries divided by 2, Loose forwards = total number of injuries divided by 3. Figure 12 illustrates the normalised injury incidence per player per position across the two tournaments. In 2019, the scrumhalf and flyhalf positions had the highest normalised injury incidence rates across both tournaments. Scrumhalves, when normalised per player, had an injury incidence of 3 (0 to 5) injuries/1000 player hours and flyhalves 2 (0 to 4) injuries/1000 player hours (Figure 12). Figure 13 shows the normalised positional injury rates per tournament. No loose forwards were injured in the Gu16W tournament.[Fig f27-2078-516x-33-v33i1a12487][Fig f28-2078-516x-33-v33i1a12487]

**Figure f27-2078-516x-33-v33i1a12487:**
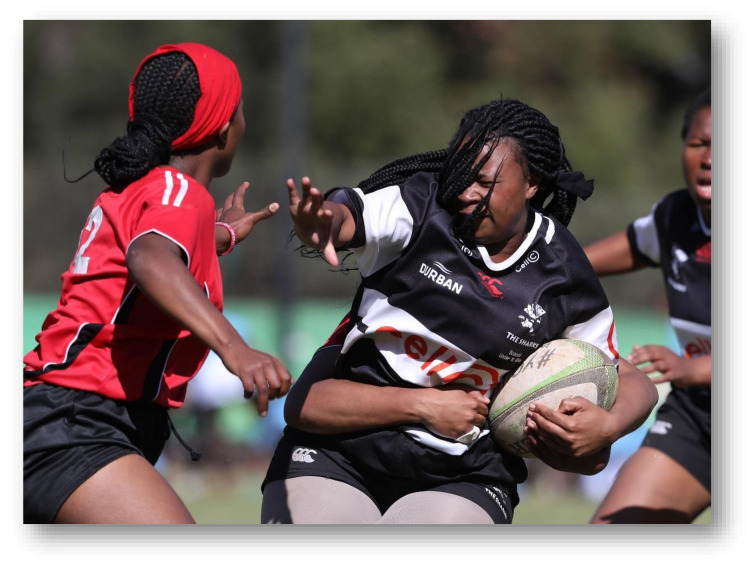


**Figure f28-2078-516x-33-v33i1a12487:**
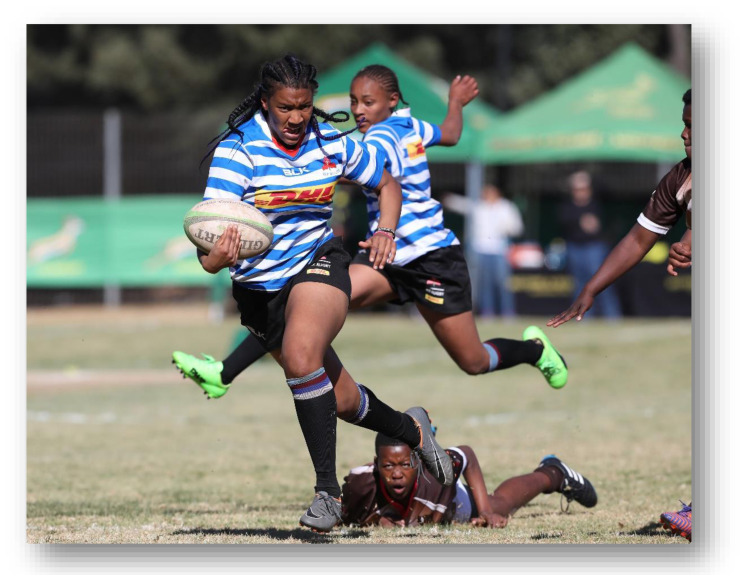


## Concussion

In 2019 there were 14 concussions. This translates to an incidence rate of 9 (4 to 14) concussions/1000 player hours and roughly one concussion for every 3 matches played. This was the second highest number of concussions recorded during the data collection period, with the highest being in 2018.

The Gu18W was the tournament with the highest concussion incidence rate of 11 (4 to 18) concussions/1000 player hours (Table 7). These data converted to 3 matches/concussion event. The Gu16W had the lower tournament concussion rate of the two tournaments, with one concussion occurring within every 5 matches played (Table 7). There were no significant differences between tournaments.

**Figure 14:**
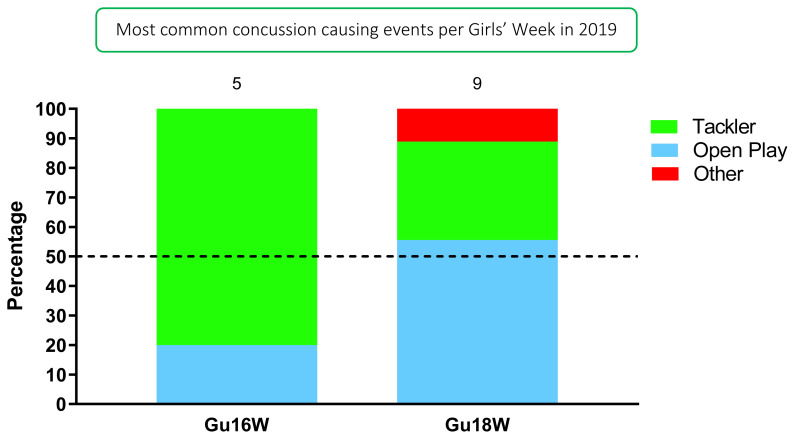
Proportion of concussions caused by the different injury events at the 2019 SARU Girls’ Youth Week Tournaments (n = 14 concussions; Gu16W = 5, Gu18W = 9).

**Figure 15:**
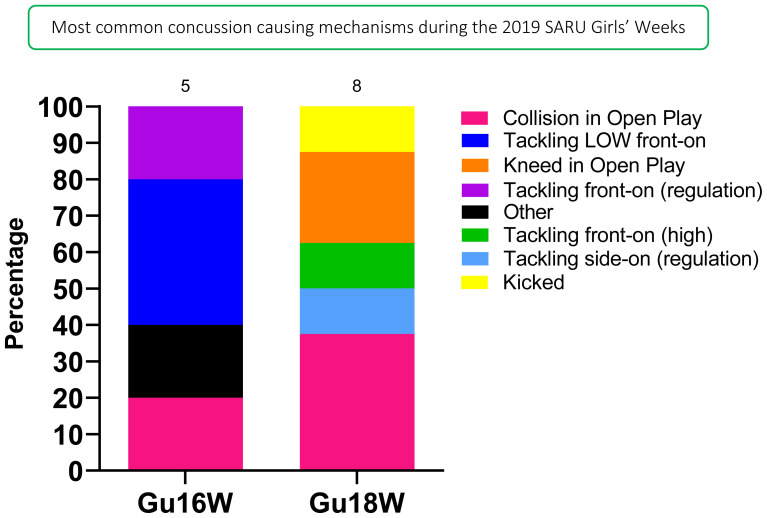
Proportion of concussions caused by the different injury mechanisms at the 2019 SARU Girls’ Youth Week Tournaments (The number above each bar represents the total number of concussions for that tournament). Missing data in 2019 = 1 case.

Tackling (50%, n = 7) contributed to the most concussions in 2019 (Figure 14). Figure 14 displays the proportion of concussions caused by the different injury events across the two tournaments in 2019. *Collision in Open Play (29%)* was the most prominent mechanism causing concussions for the combined tournament data, followed by *Tackling LOW front-on* and *Kneed in Open Play* (14%) (Figure 15).

In Gu18W, *Collision in Open Play* contributed to 3 cases recorded in 2019. Furthermore, *Tackling LOW front-on* was most prominent in Gu16W, contributing to 2 of the combined 14 cases (Figure 15).

**Figure 16:**
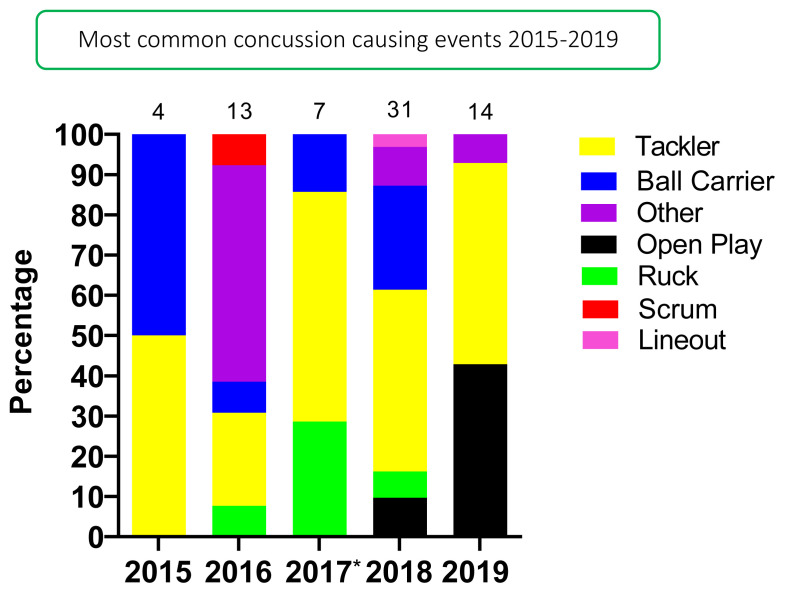
Proportion of concussions caused by the different injury events from 2015 to 2019 SARU Girls’ Youth Week Tournaments. (The number above each bar represents the total number of concussions for that year). *No Gu16W tournament was held in 2017.

Figure 16 displays the proportion of concussions caused by the different mechanisms from 2015 to 2019. The *Tackle* event was the most prominent cause of concussions between 2015 and 2019, contributing to 61% of all concussions: 44% to the Tackler, 17% to the Ball Carrier. This could possibly be due to poor tackle technique; however, further investigation is needed to verify this. Regardless, due to its high proportionate contributions in all injuries and concussions, it is apparent, that *tackling* technique is something that needs to be given more attention in training and preparation of young South African female rugby players.

The absolute number of concussions decreased since 2018, with 2019 being the first year to record no concussions to the Ball Carrier.[Fig f29-2078-516x-33-v33i1a12487]

**Figure f29-2078-516x-33-v33i1a12487:**
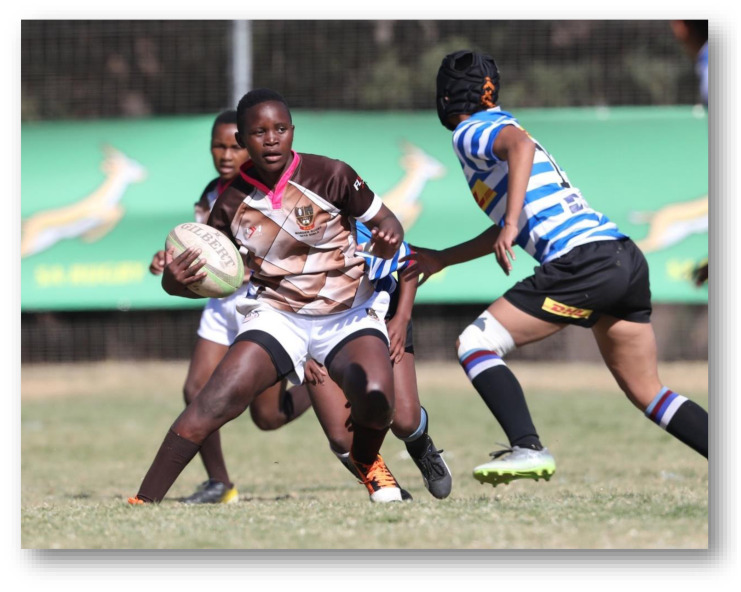


**Figure 17:**
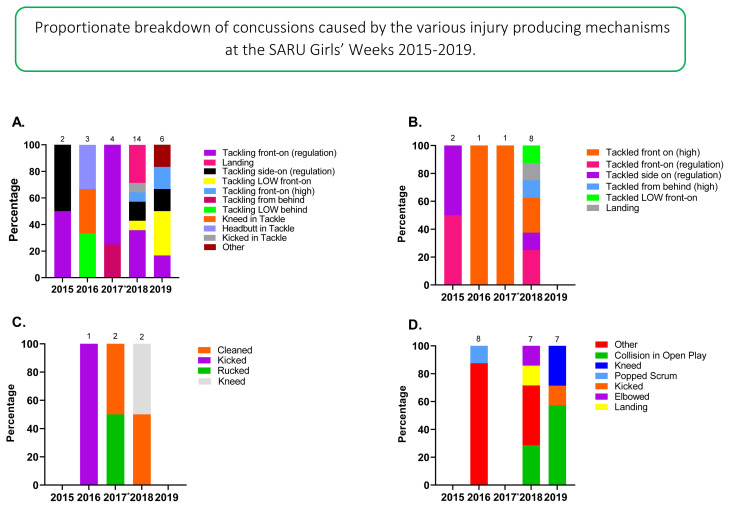
Proportionate breakdown of concussions caused by the various injury causing mechanisms at the 2015 to 2019 SARU Girls’ Youth Week Tournaments. (The number above each bar represents the total number of concussions for that year in each graph category). A. Tackler-related concussion mechanisms B. Ball Carrier-related concussion mechanisms C. Ruck-related concussion mechanisms. D. Remaining concussion mechanisms. *No Gu16W tournament was held in 2017. Missing data in 2019 = 1 case.

Figure 17 displays the proportionate breakdown of concussions resulting from the different injury causing mechanisms over the 5 years studied.[Fig f30-2078-516x-33-v33i1a12487]

**Figure f30-2078-516x-33-v33i1a12487:**
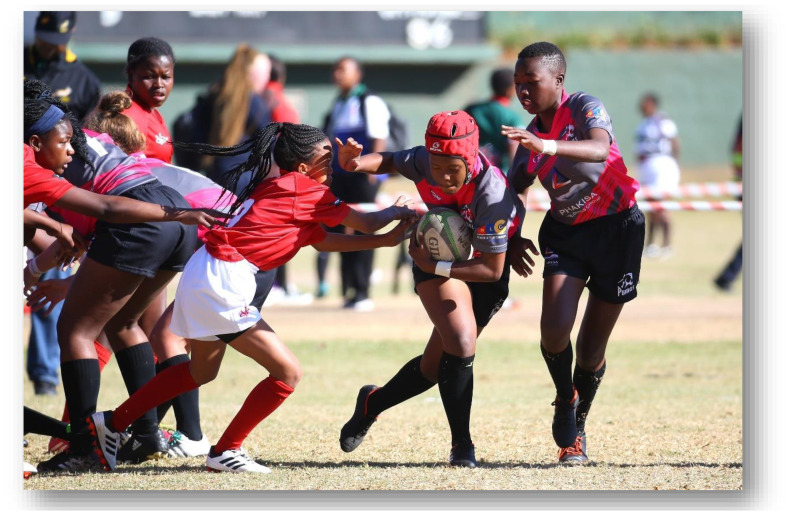


Between 2015 and 2019, 35% of Tackler-related concussions (Figure 17A) were caused by *Tackling front-on (regulation)*, 33% of Ball Carrier-related concussions (Figure 17B) were caused by being *Tackled front-on (high)* and 40% of Ruck-related concussions (Figure 17C) by being *Cleaned out*.

**Figure 18:**
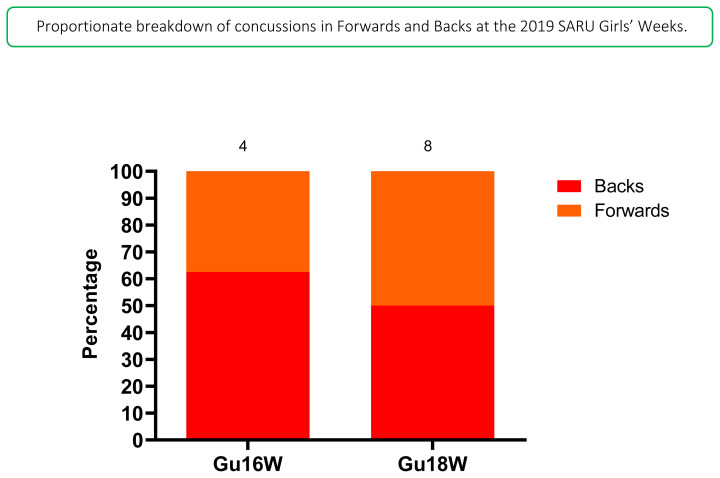
Proportionate breakdown of concussions for forwards and backs at the 2019 SARU Girls’ Youth Week Tournaments (the number above the bar represents the total number of concussions per category for that tournament). Missing data in 2019 = 2 cases.

Fifty percent (50%) of *all* concussions in 2019 occurred to backs, and also, 63% of concussions at the Gu18W were to backs (Figure 18).

**Figure 19:**
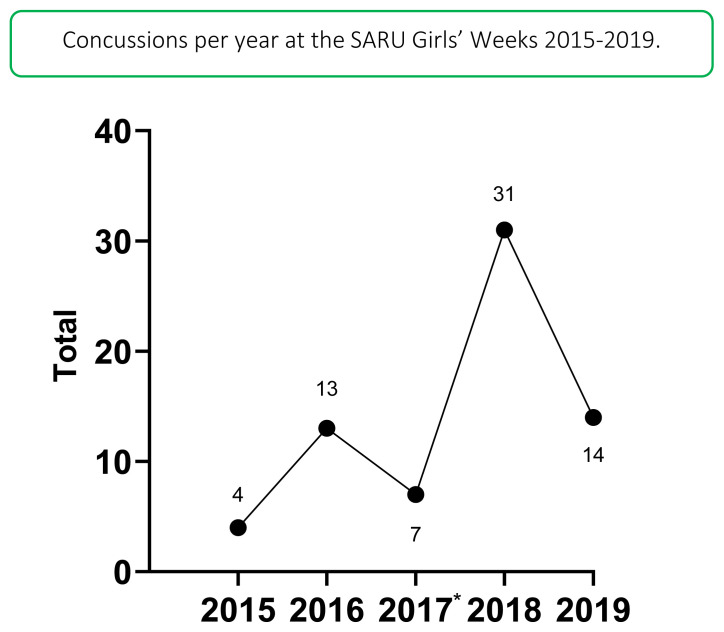
Total number of concussions per year at the SARU Girls’ Youth Week Tournaments from 2015–2019. (The number above each data point represents the total number of concussions for that year). *No Gu16W tournament was held in 2017.

**Figure 20:**
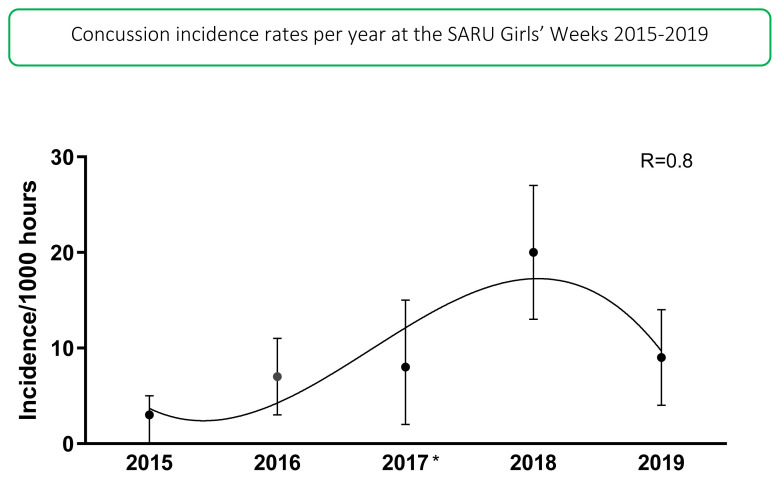
Concussion incidence rates and 95% confidence intervals/1000 player hours per year at the SARU Girls’ Youth Week Tournaments from 2015–2019. *No Gu16W tournament was held in 2017.

**Figure 21:**
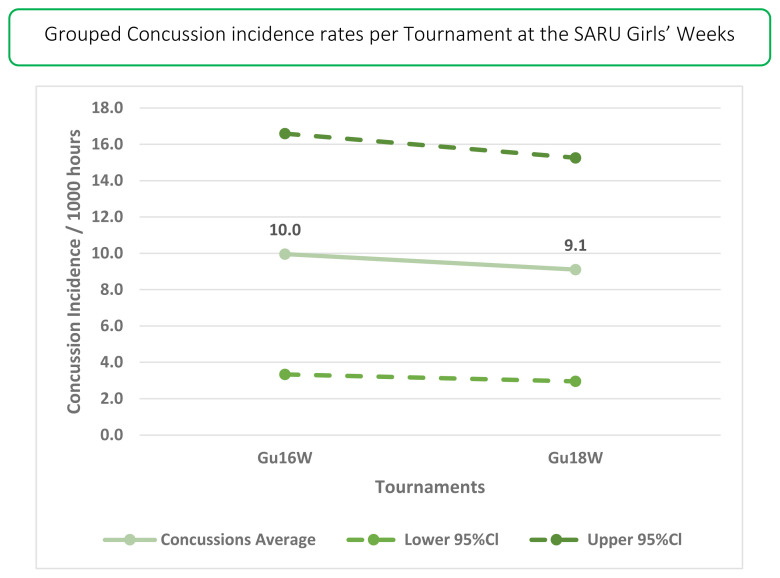
Concussion incidence rates and 95% confidence intervals/1000 player hours per SARU Girls’ Youth Week tournament from 2015 – 2019.

The total number of concussions from 2015 to 2019 for both Gu16W and Gu18W is shown in Figure 19. The corresponding rate of concussions over the same period is shown in Figure 20. The total number of concussions increases sharply from 2017 to 2018. This could be attributed to some extent to the lower number of matches played in 2017. However, the incidence of concussions, which considers the number of matches played, also shows this pattern, albeit with a more blunted increase between 2017 and 2018. Both concussion numbers and rates drop again in 2019. When combining all concussions over time per tournament (2015 – 2019), concussions and concussion rates tend to decrease from Gu16W to Gu18W (Figure 21). However, these differences are not significant.

**Figure 22:**
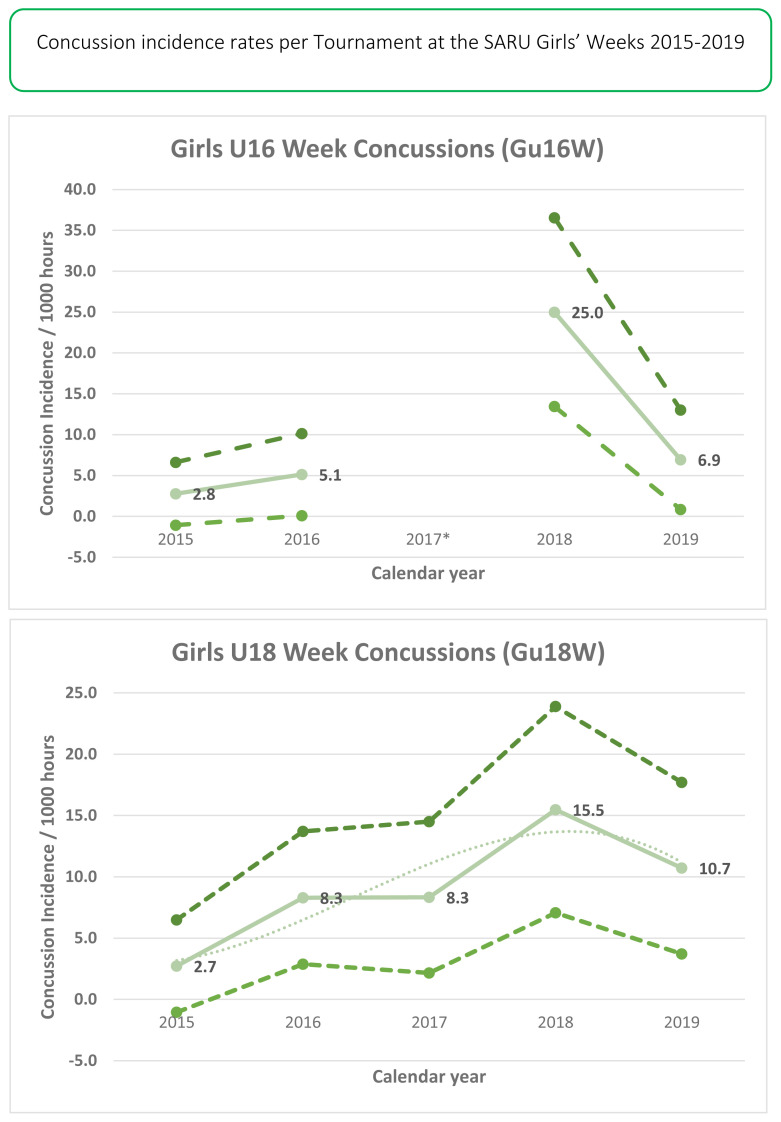
Concussion incidence and 95%CI for each SARU Girls’ Week tournament, from 2015 – 2019. The dashed grey line represents the polynomial trend. *No Gu16W tournament was held in 2017.

The changes in concussion incidence across individual tournaments (Gu16W and Gu18W) follow a similar pattern to the injury incidence trends. Concussion incidences increase from around 2015 to 2018. From 2018 to 2019 there is a sharp decrease in concussion incidence (Figure 22).

In Figure 22, there is no clear pattern of concussions for the Gu16W between 2015 and 2019. There was no Gu16W tournament in 2017, so the trendline could not be calculated with much accuracy and has therefore been excluded. Similar to the pattern for all injuries in the Gu18W, there was a spike in concussions in 2018 with a recovery in 2019. Gu18W concussions after an initial upwards trend until 2018, also lowered again in 2019. The trendline accounts for 80% of the variance in concussion incidence per 1000 player hours (R^2^ = 0.8).[Fig f31-2078-516x-33-v33i1a12487]

**Figure f31-2078-516x-33-v33i1a12487:**
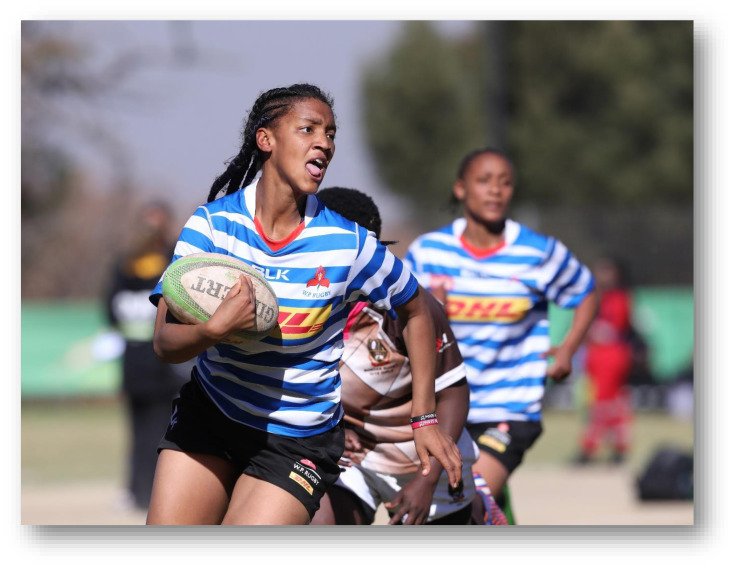


## Figures and Tables

**Table 1:** Number and injury incidence (95% CI)/1000 player hours of Medical Attention and Time-Loss injuries in the 2019 SARU Girls’ Youth Week tournaments.

	Medical Attention Injuries		Time-Loss Injuries	

	Number	Incidence	Number	Incidence
**Gu16W**	77	107 (83 – 131)	16	22 (11 – 33)
**Gu18W**	94	112 (89 – 135)	16	19 (10 – 28)

***Combined Total***	***171***	***110 (93 – 126)***	***32***	***21 (13 – 28)***

**Table 2:** Number of Medical Attention and Time-Loss injuries. Data expressed per match and per hour played in the 2019 SARU Girls’ Youth Week tournaments.

Tournament	Number of matches	Match duration (mins)	Medical Attention (injuries/match)	Time-Loss (injuries/match)	Medical Attention (injuries/hour)	Time-Loss (injuries/hour)
**Gu16W**	24	60	3.2	0.7	3.2	0.7
**Gu18W**	24	70	3.9	0.7	3.4	0.6

***Combined Tournament Average***	***24***	***65***	***3.6***	***0.7***	***3.3***	***0.6***

**Table 3:** Injury incidence (95% CI)/1000 player hours of Time-Loss injuries to the Tackler and Ball Carrier roles (within the Tackle), and the Open Play phase of play for the 2019 Girls’ SARU Youth Week tournaments.

Tournament	Tackler	Open Play	Ball Carrier
**Gu16W**	11 (3 – 19)	4 (0 – 9)	4 (0–9)
**Gu18W**	4 (0 – 8)	8(2 – 15)	2(0–6)

***Combined total***	***7 (3 – 11)***	***6(2 – 10)***	***3(0 – 6)***

**Table 4:** Injury incidence (95% CI)/1000 player hours of Time-Loss injuries at the 2019 SARU Girls’ Youth Week tournaments grouped as Joint/Ligament, Muscle/Tendon and Central Nervous System (CNS) injuries.

Tournament	CNS	Joint/Ligament	Muscle/Tendon
**Gu16W**	7 (1–13)	6 (0 – 11)	0 *
**Gu18W**	11 (4 – 18)	1 (0 – 4)	1 (0 – 4)

***Combined Total***	***9 (4–14)***	***3 (0 – 6)***	***1 (0 – 2)*** *

* Significantly lower than CNS injury types

**Table 5:** Proportion (%) and incidence (95% CI)/1000 player hours of Time-Loss injuries, grouped by body location, in the 2019 SARU Girls’ Youth Week tournaments.

	Proportion of injuries (%)	Incidence (95% CI)/1000 player hours
**Head and Neck**	69	14 (8 – 20) *
**Trunk**	3	1 (0 – 2)
**Upper Body**	9	2 (0 – 4)
**Lower Body**	16	3 (0 – 6)

* Significantly higher than Trunk, Upper Body and Lower Body injury locations

**Table 6:** Injuries grouped according to the IOC recommended categories of Tissue and Pathology types for the 2019 SARU Girls’ Youth Week tournaments. Missing 2019 data for mean time loss = 13 cases.

Tissue	Injuries	Incidence	Mean time loss

*Pathology*	n	Injuries per 1000 hours (95% CI)	Days (95% CI)
**Muscle/Tendon**	1	1 (0 – 2)	2
*Muscle strain*	1	1 (0 – 2)	2
**Nervous**	14	9 (4 – 14)	29 (10 – 47) ****
*Concussion*	14	9 (4 – 14)	29 (10 – 47) ****
**Ligament/Joint Capsule**	5	3 (0 – 6)	7 (0 – 19)
*Ligament sprain*	2	1 (0 – 3)	3 (2 – 4)
*Joint injury*	3	2 (0 – 4)	4 (1 – 7) **
**Superficial tissue/skin**	4	3 (0 – 5)	10 (0 – 25)
*Contusion (superficial)*	4	3 (0 – 5)	10 (0 – 25)
**Other injury** *	8	5 (2 – 9)	12 (0 – 29) ***

***TOTAL***	**32**	***21 (13 – 28)***	**13 (5 – 20)**

Where n = 1, mean Time-Loss reflects the total Time-Loss days. Estimated severity for Time-Loss was used from data provided by the Tournament Doctors at the venue when real-time severity was not able to be determined.

* Due to six of the cases not being finalised properly and with operational follow-up notes not being received from the treating hospital emergency units, they have been classified as ‘Other injury’, with the broader effects of the available preliminary case notes, incorporated into the ‘Other injury’ category calculations. It can however be confirmed that none of them were serious injuries, and all were confirmed as minor, precautionary cases.

** 1 case missing mean time loss data,

*** 3 cases missing mean time loss data,

**** 9 cases missing mean time loss data.

**Table 7:** Number and incidence of concussions (95% CI)/1000 player hours at the 2019 SARU Girls’ Youth Week tournaments.

Tournament	Number	Incidence	Number of matches per concussion event
**Gu16W**	5	7 (1 – 13)	5
**Gu18W**	9	11 (4 – 18)	3

***Combined Total***	***14***	***9 (4 – 14)***	***3***
